# Migration of the femoral component and clinical outcomes after total knee replacement: a narrative review

**DOI:** 10.1007/s12306-020-00690-8

**Published:** 2020-12-14

**Authors:** R. Zinno, S. Di Paolo, G. Ambrosino, D. Alesi, S. Zaffagnini, G. Barone, L. Bragonzoni

**Affiliations:** 1 Dipartimento di Scienze Biomediche e Neuromotorie DIBINEM, Università di Bologna, Via Giulio Cesare Pupilli, 1, 40136, Bologna, BO Italy; 2 Clinica Ortopedica e Traumatologica II, IRCCS Istituto Ortopedico Rizzoli, Via G.B. Pupilli 1, 40136 Bologna, Italy; 3 Dipartimento di Scienze per la Qualità della Vita QuVi, Università di Bologna, Bologna, Italy

**Keywords:** TKR, Migration, Clinical outcome, RSA, Femoral component, Cobalt-chrome

## Abstract

Loosening is considered as a main cause of implant failure in total knee replacement (TKR). Among the predictive signs of loosening, migration is the most investigated quantitative parameter. Several studies focused on the migration of the tibial component in TKR, while no reviews have been focused on the migration of the femoral component and its influence on patients’ clinical outcomes. The aim of this narrative review was (1) to provide information about of the influence of migration in femoral component of TKR prostheses, (2) to assess how migration may affect patient clinical outcomes and (3) to present alternative solution to the standard cobalt-chrome prostheses. A database search was performed on PubMed Central® according to the PRISMA guidelines for studies about Cobalt-Chrome femoral component migration in people that underwent primary TKR published until May 2020. Overall, 18 articles matched the selection criteria and were included in the study. Few studies investigated the femoral component through the migration, and no clear migration causes emerged. The Roentgen Stereophotogrammetric Analysis has been mostly used to assess the migration for prognostic predictions. An annual migration of 0.10 mm seems compatible with good long-term performance and good clinical and functional outcomes. An alternative solution to cobalt-chrome prostheses is represented by femoral component in PEEK material, although no clinical evaluations have been carried out on humans yet. Further studies are needed to investigate the migration of the femoral component in relation to clinical outcomes and material used.

## Introduction

Total knee replacement (TKR) represents a valid solution for the treatment of end-stage knee osteoarthritis. With the right indications and a reliable and reproducible surgical technique, TKR has an average lifetime of nearly 20 years with in vivo use before revision surgery becomes a necessity [1]. A recent systematic review suggests that the rate of survival at 25 years of TKR is 82% [2]. Anyway, there is still a considerable percentage of TKR failure whose consequent revision surgery might occur earlier than 20–25 years.

There are the causes that can lead to TKR failure: the most frequent is aseptic loosening, followed by infection, unexplained pain, wear, instability, and periprosthetic bone fractures [3–6]. Some of these causes seem to be favored by stress shielding. Indeed, stress shielding is an inevitable phenomenon occurring mainly in the first year after TKR [7]. It is caused by the different stiffness of bone and prosthetic implant, with the latter being nearly one order of magnitude stiffer than the former. It has been demonstrated that stress shielding reduces the load at the bone–prosthesis interface and leads to a gradual bone remodeling and osteolysis which, in turn, can lead to aseptic loosening of the implant or, to a lesser extent, can weaken the bone such that it will fracture [8]. According to Parchi et al. [7] stress shielding causes a constant decrease of periprosthetic bone mineral density (BMD), especially at femoral level, mainly during the first 3–6 months following surgery.

However, aseptic loosening can also be caused by wear, fixation and/or migration of implant components.

As far as clinical symptoms are concerned, patients presenting with loosening of TKR components and requiring surgery might be completely asymptomatic or present the insidious onset of knee pain, most commonly following a prolonged pain-free interval after the index procedure [9]. Considering the variability in clinical presentation and the need for a prompt diagnosis, migration was deemed a useful predictor for late-term risk for revision of TKR [10]. Indeed, migration has been revealed to be able to predict implant failure, even before clinical symptoms appear. Therefore, migration is advised as a key marker for the quality of a TKR.

Understanding the biological behavior of the bone in contact with the prosthetic surface and how it can affect implant survival and clinical outcomes, might lead to the development of newer designs and materials (e.g., with stiffness closer to the one of the bones) that could provide significant benefits to improve function and survival rate after TKR.

Several studies focused on the migration of the tibial component in TKR, and reviews have already been performed on this topic [10]. No literature reviews have been focused on migration of the femoral component and its influence on patients’ clinical outcomes.

Therefore, the purpose of this narrative review was to provide (1) information about the influence of migration in the femoral cobalt-chrome (CoCr) alloy components routinely used in TKR, (2) to assess how this migration may affect patient clinical outcomes, and (3) to present alternative solutions that could replace materials traditionally used in joint prostheses, overcoming the issues related to the mechanical properties.

## Material and methods

### Data sources

An electronic database search was performed on August 1, 2020, using PubMed Central® to identify articles concerning general CoCr femoral component micromotion in people that underwent primary TKR and how it affected the patients’ clinical outcomes.

### Search terms

The terms and keywords used for the literature research were (‘femoral') OR (‘femur’) AND (‘micromotion') OR (‘migrat*’) OR (‘sink*’) OR (‘loss’) OR (‘loos*’) AND ('total knee arthroplasty’) OR (‘TKA’) OR (‘total knee replacement’) OR (‘TKR') located within the title and/or abstract.

### Study selection process

**Figure 1.**
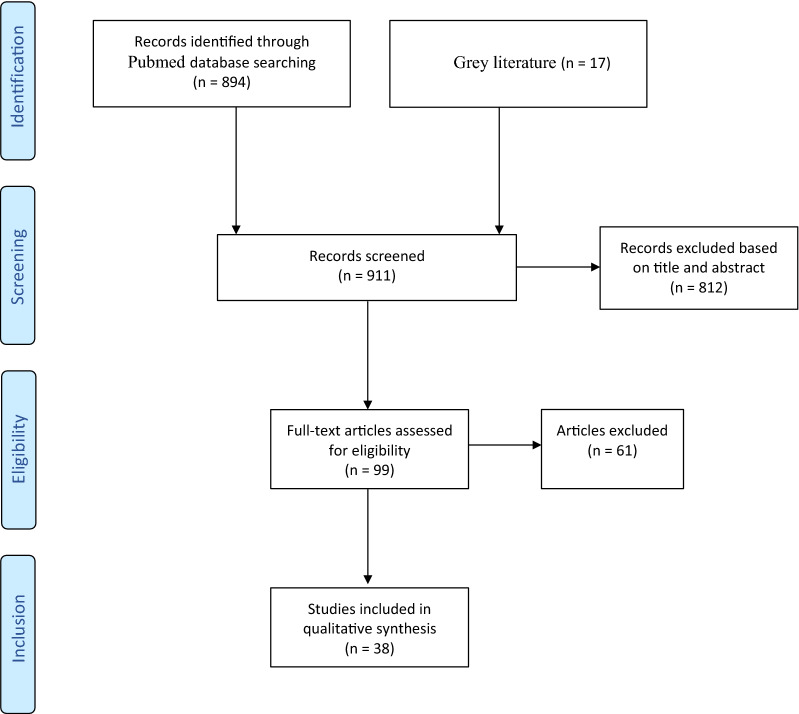
Flow chart of the narrative review according to the PRISMA guidelines

All articles published until August 2020 were included in this review. During the screening procedure, only full-text available items, written in English language, were considered; pre-clinical and ‘other animal’ studies were included; moreover, reviews were added to the list. Subsequently, the authors further screened title and abstract of the papers, in order to exclude the irrelevant ones for this review. Then, the authors full-screened the remaining papers to leave out those not concerning femoral micromotion analysis, while papers concerning femoral components materials alternative to most used CoCr were included. In the end, 21 papers were included in the review. Furthermore, 17 papers (gray) mentioned in the selected works were added, since they did not appear in the first screening (Figure 1).

## Results

### Causes and evaluation methods of migration

Only few studies assessing migration of the femoral component were retrieved, in contrast to the numerous studies assessing the migration of the tibial component (Table 1). No clear evidence of migration causes emerged from the analysis. However, a possible cause of migration could be related to bony fixation. Indeed, the lack of bony fixation may cause the implant to become unstable and migrate [1]. Moreover, factors such as low mineral density, bone remodeling, and reabsorption might lead to implant migration [11].

**Table 1.** Summary of literature related to migration of femoral component and clinical outcomes in total knee replacement

Authors	Year	Type of study	Aim	Instruments	Study subjects	Outcome	Results	Conclusions
Howard et al. [1]	2014	Observational study	To assess the morphology of the fixation interfaces in femoral component	Radiograph	Nineteen fresh-frozen knees with TKR postmortem: 16 cemented 2 cementless 1 partially cementless	Femoral component fixation (contact fraction)	Total contact friction:10.3% cemented, 10.65% cemented press-fit, 6.5% press-fit	Minimal fixation seems necessary for long-term success of TKA femoral components
Ruiter et al. [4]	2017	Finite element study	To compare PEEK and CoCr implants for mechanical performance and fixation	Finite element simulations of level gait	CoCr PEEK Intact knee (controls)	Stresses for (1) the femoral component (2) the cement mantle	Peak compressive stresses: CoCr 75 Mpa; PEEK 34 MPa Bone strain energy density distribution higher in CoCr	Stress for the cement mantle: similar for PEEK and CoCr femoral component reduced stress shielding in PEEK
Parchi et al. [7]	2014	Review	To analyze changes in periprosthetic bone	PubMed	Total knee replacement, total hip replacement	Periprosthetic bone mineral density	Constant decrease of periprosthetic bone mineral density in first 3-6 months	Femoral bone loss after TKA seems to be related to the stress shielding
Fraser et al. [9]	2015	Review	To evaluate wear and osteolysis	Not specified	Total knee replacement	Wear Rates and Osteolysis Clinical Evaluation for Osteolysis Treatment Options	Rate of particulate debris dependent on component’s design, positioning, and material properties Patients with osteolysis can be completely asymptomatic	Wear rates can be reduced by achieving proper alignment and component positioning with an index procedure and by using modern highly cross-linked polyethylene inserts
Pijls BG et al. [10]	2018	Systematic review and meta-analysis	To evaluate the early and long-term migration of tibial components of all known RSA studies	Medical librarian PubMed, Embase Web-of-Science Cochrane Library	2470 patients with TKR	MTPM	6 months - 1 year = 0.04 mm MPTM 1 year - 2 years = 0.04 mm MPTM1 year: Cemented 0.44 mm / Cementless 1.09 mm	First evaluation of the safety (i.e., implant-bone fixation) of the implant at 6 months
Henricson et al. [11]	2019	Randomized controlled trial	To study the migration of the femoral component and clinical outcomes up to 10-year follow-up	Radiostereophotogrammetric analysis	41 patients:19 Cem, 22 cem.less 23 women, 18 men Age: under 60years	MTPM	Cemented 0.85 mm (median) Cementless 1.44 mm (median) No differences in migration or clinical results at 10 years	Annual migration of 0.1 mm seems compatible with excellent long-term performance
Gao et al. [14]	2009	Prospective randomized controlled study	To compare the magnitude and pattern of migration of cemented versus uncemented fixation of the femoral component by using radiostereometry	Radiostereophotogrammetric analysis	41 patients (22 cemented, 19 uncemented) younger than 60 years	MPTM	6 week: Cem 0.41 (0.20–0.71), Cem.less 0.36 (0.31–0.46) 3 month: Cem 0.45 (0.24–0.87), Cem.less 0.53 (0.36–0.67) 12 month: Cem 0.62 (0.39–0.96), Cem.less 0.63 (0.39–1.11) 24 month: Cem 0.72 (0.38–1.62), Cem.less 0.87 (0.47–1.10)	Uncemented and non-HA-coated femoral component may behave equally as well as a cemented one in the long term.
Seehaus et al. [15]	2009	Experimental study	To evaluate the experimental accuracy and precision of the MBRSA method for four different, but typical prosthesis geometries that are commonly implanted	Radiostereophotogrammetric analysis	1 femur, 1 tibia, and 2 hip (argo-TEP and Antea)	Translation and rotation	MBRSA in-plane: better than -0.034 to 0.107 mm translation, and -0.038 to 0.162 deg out-of-plane: better than − 0.217 to 0.069 mm translation, and − 1.316 to 0.071 deg	MBRSA method can be used with many common implant geometries, and the method could lead to a wider application of the RSA for investing clinical implant fixation that has been possible to date.
Järvenpää et al. [19]	2014	Prospective study	To assess long-term periprosthetic BMD changes after TKR in obese and nonobese patients	DEXA	69 TKR in 61 patients	Bone mineral density	Average bone loss at 7 years: 17.6% in anterior, 30.7% in central, 17.6% in posterior, 22.2% in total metaphyseal ROIs, 10.3% in diaphyseal ROI	Bone loss is likely caused by the stress shielding and immobilization in the first postoperative phase
Berahmani et al. [20]	2017	Experimental and pre-clinical analysis	To evaluate the primary stability of the Attune cementless femoral components, and compared it against a conventional implant under simplified gait and deep knee bend loads	DIC	6 pairs of femur	BMD Micromotion	Attune: 126 mg/cm2 BMDLCS: 136 mg/cm2 BMDGaitAttune 32 µmLCS 71 µmDKBAttune: 55 µmLCS: 83 µm	Micromotions of Attune were significantly lower than LCS under both loading conditions BMD was only a significant factor affecting the micromotions under simplified gait loading
Schroder et al. [21]	2001	Prospective study	To report long-term results with TKR in an unselected series of patients with osteoarthrosis and rheumatoid arthritis	Radiograph Questionnaire	114 patients (cementless)	Alignment Clinical score Survival rate	Alignment 10-year follow-up: 18 Varus (<2°), 37 Neutral (<2°) Excellent knee score: Preop (0%), 3 years (70%), 7 years (65%), 10 years (76%) Survival rate: 97.1 %	Cementless insertion of a nonmodular, porous-coated TKA resulted in a long-term durable bone-prosthesis interface.
Park et al. [23]	2011	Prospective randomized study	To evaluate the clinical and radiological results of the NexGen TKR cemented or cementless implanted bilaterally in the same patient	Radiograph Questionnaire	50 patients (100 knees); 39 women and 11 men, mean age of 58.4 years (51–67)	Radiological results Knee score Function score Walking distance ROM	KSSc: 96.2 Cem, 97.7 Cem.less KSSf: 85.8 Cem, 88.1 Cem.less Unlimited walking distance: 82% Cem, 82% Cem.less ROM supine: 124° Cem, 128° Cem.less Radiolucent line < 1 mm: 4% Cemented, 6% Cementless	No advantage of cementless over cemented components in total knee replacement
Wang et al. [24]	2020	Systematic review and meta-analysis	To evaluate the optimal fixation mode in TKR for young patients.	PubMed Embase Medline Web of Science full Cochrane Library	510 Knees:255 Cemented255 Cementless	Functional outcomes KSS ROM Radiolucent linesAseptic loosening Total complications Reoperation rate	Radiolucent line < 1mm: 18.4% Cem, 9.8% Cem.less KSSf: Higher in Cem.less KSSc: Higher in Cem.less KSSpain: Higher in Cem.less ROM recovery: Higher in Cem.less	Cementless TKR was substantially superior to cemented TKR in young patients
Han et al.[25]	2007	Retrospective study	To determine whether the increased loading in the knee during deep flexion substantially increases wear of the insert or loosening of components.	Radiograph Questionnaire	72 knees of 47 patients (44 women, 3 men)	Radiolucent Clinical and functional score Survival rate	HSS pain: Preop 5.5, Postop 28.5 HSS function: Preop 14.6, Postop 20.1 Survival rate: Revised 21% (15), Well-fixed 79% (57)	The loosened femoral components were found to migrate into a more flexed position, but no migration was detected in the well-fixed group.
Nilsson et al. [26]	1995	Prospective randomized study	To evaluate the relative micromotion of cemented and cementless femoral components using RSA	RSA Questionnaire	33 knees (29 primary osteoarthritis , 4 secondary osteoarthritis)	MPTM Clinical outcomes	MPTM: 0,89 ± 0,08 mm Cementless; 0,88 ± 0,16 mm Cemented HSS: 89 Cementless; 90 Cemented	No differences in fixation of the femoral component cemented and cementless 2 years
Nieuwenhuijse et al. [27]	2013	RSA study	To compare the migration and clinical outcomes of high flexion TKR fixed and mobile bearing with conventional	Model-based RSA Questionnaire	42 knee	MPTM Clinical outcomes	Migration: no differences between groups KSS: 34.8 ± 11.7 LPS-Flex Mobile 38.4 ± 18.9 LPS-Flex Fixed 33.7 ± 10.0 LPS Mobile 32.4 ± 12.8 LPS Fixed KSS function: 23.2 ± 17.7 LPS-Flex Mobile35.8 ± 24.7 LPS-Flex Fixed27.5 ± 25.5 LPS Mobile33.8 ± 20.7 LPS Fixed	Migration of the LPS high flexion TKR was comparable with those of the LPS conventional TKR and independent of the bearing type used
Ruiter et al. [32]	2017	Finite Element study	To investigate the mechanical response of a PEEK TKR device during a deep squat	A finite element model of a TKR subjected to a deep squat loading condition	CoCr PEEK Intact knee (controls)	Stress in femoral component Stress in cement mantle Stress shielding	Femoral component: 60MPa (145°) CoCr, 30MPa (145°) PEEK Cement mantle: 12MPa (120°) CoCr, 24MPa (145°) PEEK Stress shielding: similar in PEEK implant and the intact bone remodeling stimulus	PEEK femoral implant is strong enough to endure high demand loading and has potential for periprosthetic bone stock retention
Rankin et al. [33]	2016	Preliminary Laboratory Study	To investigate whether PEEK TKR femoral component induces a more physiologically normal bone strain distribution than a CoCr component	Digital Image Correlation (DIC) technique	CoCr PEEK Intact knee (controls)	Strain distribution in intact femur, CoCr and PEEK	Strain shielding in CoCr implant was lower than the intact case(* p* = 0.014) Strain in PEEK implant deviated less from the intact case with no difference (*p* = 0.231)	PEEK femoral component could transfer more physiologically normal bone strains with a reduced stress shielding effect
Du et al. [34]	2018	Randomized controlled trial	To gather preliminary evidence on the performance and safety of a cemented PEEK-based TKR	Radiographic examination (4, 12, and 24 weeks postoperatively)	15 Adult goats: 10 experimental 5 control	Prosthesis condition Loosening Radiolucent line	Decreased BMD at 12 weeks (6%) compared to the controls Radiographic examination: no evidence of implant fracture, insert protruding, prosthesis loosening, or sinking during the 24 weeks (except 1 case of prosthesis dislocation)	PEEK device in a goat model was feasible and safe
Xiang et al. [36]	2013	Systematic review	To gather and analyze information regarding the clinical outcomes and reach a definitive conclusion about the use of ceramic femoral components	MEDLINE EMBASE Cochrane ClinicalTrials.gov databases	1245 Patients and 1438 Knees	Clinical outcomes	Clinical outcomes of Ceramic TKR improved: Range of motion Range of flexion HSS scores KSS scores	Ceramic TKA implants show similar postoperative clinical results and survival rate compared to metal ones
Cristofolini et al. [38]	2009	Experimental study	To test in vitro whether ceramic TKR femoral components are more prone to mechanical loosening than metal ones	Knee simulator (6 degrees of freedom)	2 Cemented prosthesis (1 ceramic vs 1 metal)	Inducible migration Permanent migration	Inducible micromotions: Metal 0.010–0.200 mm (range), Ceramic 0.023–0.162 mm (range) Permanent micromotion: Metal − 0.021 to − 0.438 mm (range), Ceramic − 0.279 to +0.201 (range)	No difference was observed for the inducible micromotion, permanent micromotion or amount of damage between both prosthesis

The quantity of migration has been mostly assessed through the maximal total point motion (MPTM). The MTPM is the unit of measure for the largest 3D migration of any point on the prosthesis surface [12]. The calculation of MTPM is mainly performed through Roentgen Stereophotogrammetric Analysis (RSA). There are two different methods: on the one hand, the manual marker-based; on the other hand, the semi-automatic CAD model-based [10]. Both methods are suitable for in vivo measurement of implant migration in clinical research studies concerning the TKR [13]. Indeed, RSA measurements are claimed to have a high prognostic precision in early detection of potential late occurring aseptic loosening [14, 15]. Moreover, RSA allows the calculation of the “inter-marker distance” parameter, which can be seen as an index of material deformation within the different districts of a prosthetic implant (e.g., for the TKR, condyles and shield) [16]. RSA technique has been successfully used also in other joint surgery contexts and in presence alternative material solutions, e.g., in hip prosthesis to assess migration and material deformation of less stiff stems [17] and in spinal arthrodesis to predict lumbosacral stability of carbon fiber-reinforced cages [18].

Since the migration is linked to bone remodeling, measurement of bone density is crucial. Therefore, the use of dual-energy X-ray absorptiometry (DEXA), evaluating the bone density, could be a useful tool. Indeed, DEXA analysis could be used also in the assessment of bone remodeling of the femoral condyles after TKR [7]. Three studies show a dominating tendency toward decrease in tibia and femur bone mineral density (BMD) after the implantation of TKR [7–19]. However, BMD was shown to be an effective tool only in some specific loading conditions, as stated in a pre-clinical cadaveric study [20].

### Quantification of migration and patients’ outcomes

Due to the lack of studies regarding the femoral component, no migration thresholds suggesting short- and long-term survival of the femoral component prosthetic implants were retrieved.

Migration patterns must be evaluated through at least three-times assessments, one at baseline and two follow-ups within the first 2 years. For the tibial component, the most frequently reported follow-up time for MTPM evaluation was 1 year [10]. Nevertheless, the literature reported other time intervals, as well: 6 weeks, 3 months, 6 months, 2 years, 5 years, and 10 years [10, 14].

Three RSA studies have shown that loosening can be concretely assessed in the early postoperative period [12–22]. Henricson et al.[11] reported a displacement of the femoral component MTPM of 0.10 mm per year for cemented implant and 0.09 mm per year for the cementless implant, throughout a 10-year follow-up evaluation. Few studies correlated the amount of migration with the patients’ outcomes. Henricson et al.[11] suggested that an annual migration of 0.10 mm seems compatible with good long-term performance and good clinical and functional outcomes at 10-year follow-up [11]. Gao et al [14] found the same clinical and radiological results with patients younger than 60 years old.

These results are in accordance with Park et al.[23], who evaluated the clinical and radiological results comparing the identical cemented or cementless TKR design, implanted bilaterally in the same patient. They showed that after 14 years from surgery, the survival rate was 100% for both femoral components. Moreover, no differences were found in the outcomes like KSS, Western Ontario and McMaster Universities Arthritis Index (WOMAC), Visual Analogue Scale (VAS), range of movement (ROM), and radiological results.

On the contrary, Wang et al.[24] reported that the cementless group had better KSS-function and KSS-pain, better ROM recovery, and fewer radiolucent lines (<1mm) than the cemented one, in a systematic review with >500 knees comparing postoperative outcomes of fixation in primary TKR for young patients (<65 years). Hence, they suggested that cementless TKR was substantially superior to cemented TKR in young patients [24].

A further study showed that the migration strongly affects TKR outcomes: in revised TKR with high-flexion design, the loosened femoral components migrated into a position of increased flexion from a mean of 4° immediately postoperatively to a mean of 7° at the final review, whereas no migration into flexion was observed in the control TKR group [25].

Two more RSA studies compared different TKR designs at 2- [26] and 5-years [27] follow-up. The former did not find differences in MTPM between cemented (0.88 mm) and cementless (0.89 mm) TKR designs. For both groups, the MTPM was higher in the posterior condyles. Peculiarly, the only one case of revision was predicted by an MTPM up to 4.1 mm at 12 months. The authors further stated that such loosening could be caused by trabecular microfractures occurring some millimeters away from bone–implant interface, in presence of bone softened due to stress-shielding [27]. The latter study did not find differences between four TKR designs (high/conventional flexion with fixed/mobile bearing). The MTPM was always about 1 mm. The only case of loosening presented with early migration over 2 mm within the first 3 months and reached up to 12 mm at one year.

### Alternative solutions to standard CoCr implants

The vast majority of TKR implants found in the present review were made of CoCr alloy. As evidenced from the literature search, nonsignificant migration differences were found between different TKR designs. Therefore, implant loosening might be influenced by further factors, e.g., the material properties of the component. The two main alternatives found in the literature regarded the use of nonmetal materials, i.e., the polyethylene and the ceramic. The former was found either in terms of all-polyethylene or polyetheretherketone (PEEK). Polyethylene is less stiff than CoCr alloys and is therefore claimed to reduce the stress shielding at bone–implant interface [28]

All polyethylene material was only used in tibial components in TKR, and the MPTM has been evaluated with respect to the metal-backed ones. The most recent studies [28–31] underlined a comparable amount of migration and risk of loosening between the two different materials. Furthermore, Norgren at al. [28] found a greater internal–external rotation in metal-backed tibial components and ascribed it to a greater stiffness of the latter.

Only few pre-clinical studies reported the use PEEK material in TKR context. Such material has already been used in different surgical scenarios, such as spinal and cranio-maxillofacial surgery, and it has shown a good level of rigidity, durability, and biocompatibility [4]. A finite element study analyzing the prosthetic implant loads during a gait cycle predicted that the performance of the PEEK femoral component would not be inferior to the CoCr femoral implant [4]. They also suggested that PEEK implant could cause a lower periprosthetic stress shielding compared to a standard implant [4].

The same type of analysis was performed during a high demanding activity (deep squat). PEEK implant showed higher compressive and lower tensile cement stress, thus demonstrating no increased risk of failure compared to the CoCr implant [32]. Furthermore, in the same study, the PEEK component showed bone strains more similar to the intact bone than the CoCr component [32].

Rankin et al.[33] used a digital image correlation (DIC) technique to evaluate bone strain distribution of the PEEK femoral component. Such prosthesis produced a bone surface strain field closer to that of the intact bone case. This further demonstrates that the reduced stiffness of PEEK implants compared with CoCr has the potential to reduce stress shielding and the risk of aseptic loosening, hence potentially improving long-term bone preservation [33].

This type of prosthesis has been tested on animal in vivo models, as well: Du et al. [34] demonstrated that cemented PEEK knee replacement devices in a goat model are feasible and safe, as on the basis of radiographic images, there was no evidence of implant fracture, insert protruding, prosthesis loosening, or sinking during the 24 weeks, except for one case of prosthesis dislocation, that did not affect its activity as soft tissue could maintain the stability of the joint. Moreover, the goats returned to perform activities like squatting, standing up, jumping, and running.

Although PEEK material for TKR demonstrated promising results in pre-clinical investigations, no studies have been carried out in vivo on human patients. Therefore, its dependability in a clinical context is yet to be confirmed. However, if roughly equating the two polyethylene materials (all polyethylene and PEEK), similar migration results could be argued in vivo for a femoral PEEK component.

Ceramic components are claimed for the higher biocompatibility, durability, and resistance to scratching with respect to CoCr alloy [35]. Indeed, ceramic prosthetic implant was used in the TKR procedure with excellent long-term joint function and survival [36]. A prospective study published in 2013 investigated the short-term outcomes of the ceramic femoral component TKR and found comparable results to the metal femoral TKR [37]. Furthermore, an in-vitro study published in 2008 by Cristofolini et al. [38] investigated migration of CoCr and ceramic femoral component under cycle loadings and concluded that no sign of loosening nor significant differences were present between the implants. Therefore, this study underlined that ceramic femoral component is not mechanically inferior to a standard CoCr. Nevertheless, no recent studies (less than 10 years) investigating migration on ceramic components were retrieved in the present review.

## Conclusion

Only a limited number of studies evaluated micromotion of the TKR femoral component. There is no total agreement regarding the migration causes; at the same time, there are contrasting opinions about patients’ clinical outcomes after surgery. At the present time, the RSA technique is the most commonly used, as well as the most accurate tool to evaluate migration. Indeed, it is recognized by the scientific literature as an instrument to predict the stability and the lifetime of the prosthetic implant, both for femoral and tibial components.

Furthermore, the study raised up possible alternative solutions, such as polyethylene and ceramics. Though the latter showed good long-term results, no recent studies were retrieved (less than 10 years). This aspect could be symptomatic of an obsolescence of such alternative. PEEK material seems a suitable solution because of reduced material stiffness, which may lead to a limited stress shielding [32]. However, further studies on patients are needed to evaluate the benefits and long-term survival of such alternative in a real clinical scenario.

Given the successful use of RSA for the assessment of migration and material deformation in presence of alternative materials in other body districts, such application could be extended to a TKR context as well.
